# Botulinum Toxin Paves the Way for the Treatment of Functional Lower Urinary Tract Dysfunction

**DOI:** 10.3390/toxins12060394

**Published:** 2020-06-14

**Authors:** Hann-Chorng Kuo

**Affiliations:** Department of Urology, Hualien Tzu Chi Hospital, Buddhist Tzu Chi Medical Foundation, and Tzu Chi University, Hualien 970, Taiwan; hck@tzuchi.com.tw

**Keywords:** botulinum toxin, functional urological disorders, pain, inflammation, overactive bladder, neurogenic detrusor overactivity, interstitial cystitis, chronic pelvic pain syndrome

Botulinum toxin A (BoNT-A) is a potent protein that can selectively modulate neurotransmission from nerve endings, resulting in the blocking of neurotransmitter releases and causing muscular paralysis. Detrusor overactivity (DO) can be suppressed by intravesical BoNT-A injection, which results in a reduction in detrusor pressure and decreased urgency urinary incontinence and daily frequency. Recent evidence has also revealed that BoNT-A has a mechanism acting on sensory receptors and an anti-inflammatory effect, providing a chance for physicians to treat refractory interstitial cystitis/bladder pain syndrome (IC/BPS), chronic prostatitis (CP), and chronic pelvic pain syndrome (CPPS). The injection of BoNT-A into the urethral sphincter results in a reduction of urethral resistance and improves voiding efficiency in neurogenic and non-neurogenic urethral sphincter dysfunction.

In lower urinary tract dysfunction (LUTD), BoNT-A has recently received regulatory approval for the treatment of adult patients with neurogenic DO due to spinal cord lesions, multiple sclerosis, and idiopathic overactive bladder syndrome (OAB). Although unapproved, BoNT-A has been widely used to treat patients with neurogenic or non-neurogenic voiding dysfunction, and male lower urinary tract symptoms (LUTS) due to benign prostatic hyperplasia (BPH) or bladder-neck dysfunction. Other situations in which urologists have applied BoNT-A in recent decades include IC/BPS, bladder oversensitivity, and CP/CPPS.

This Special Issue gathers 14 articles which focus on recently published research articles and clinical trials on the pathophysiology and therapeutic effects of BoNT-A on LUTD. The basic science which supports the clinical application of BoNT-A on LUTD is also covered in this Special Issue. Technical points, adverse events, patients’ satisfaction, and their adherence to this novel treatment are also involved. Liao et al. present the mechanism of action of botulinum toxin A in the treatment of functional urological disorders [1]. They review recent reports on the effect of BoNT-A on functional urological disorders. Evidence has shown that BoNT-A not only affects the release of neuropeptides from motor nerve endings, but also connects the sensory nerve to the central nervous system. Inflammation in the central nervous system can also be reduced after BoNT-A treatment [1].

BoNT-A has been widely applied in the treatment of functional urological diseases, such as OAB, neurogenic DO, IC/BPS, and CP/CPPS. In the scope of OAB treatment, Wang et al. review the pharmacological mechanism of diabetes mellitus-associated OAB and its treatment with BoNT-A. They report that adverse events after BoNT-A injection are common in patients with diabetes mellitus and should be cautiously monitored [2]. In previous studies, it was suggested that detrusor hyperactivity, impaired contractility [3], frailty, and medical comorbidities [4] were associated with a less effective response to BoNT-A treatment and an increased risk of adverse events. Wang et al. [3], and Liao et al. [4] carried out comprehensive reviews and clinical reports of the treatment outcome in these patient cohorts. Chen et al. review the therapeutic efficacy of BoNT-A delivered using various approaches such as protamine sulfate pretreatment and low energy shock wave to increase urothelial permeability, and liposomes to create a carrier for the transportation of BoNT-A [5]. Because BoNT-A has dual motor and sensory effects, the reduction of the expression of TRPV1 and P2X3 on suburothelial sensory afferents can be achieved in patients treated with detrusor BoNT-A injections. Jiang et al. review the pathophysiology, therapeutic mechanisms and treatment effects of BoNT-A on sensory bladder disorders, from bench to bedside [6]. Lo et al. compare the efficacy of BoNT-A by meta-analysis, sacral neuromodulation, and peripheral tibial nerve stimulation in the management of OAB. Among these treatment modalities, BoNT-A resulted in higher complications including urinary tract infection and urinary retention [7].

Although the actual pathophysiological mechanism of the action of BoNT-A has not been completely demonstrated, an anti-inflammation effect might be the predominant therapeutic mechanism for bladder hypersensitivity, IC/BPS, CP/CPPS, and LUTS/BPH. Yeh et al. comprehensively review the animal models of mechanisms of BoNT-A in the inhibition of neurotransmitter release, the reduction of stretch-related visceral pain, and anti-inflammatory effects on the bladder urothelium [8]. Jhang further reports the possible mechanisms and practical issues in BoNT-A treatment for patients with IC/BPS [9]. Wang et al. report the predictive factors for a satisfactory treatment outcome of BoNT-A on IC/BPS. They found that patients with a maximal bladder capacity of ≥760 mL and a glomerulation grade of 0 or 1 after hydrodistention frequently had a satisfactory outcome [10]. Interestingly, the adverse events of BoNT-A are different between patients with IC/BPS and those with OAB. Kuo et al. previously found that the bladder contractility of OAB patients is more susceptible to BoNT-A, which might reflect the different mechanisms of action of BoNT-A on bladder dysfunctions [11]. Regarding the role of BoNT-A for chronic pelvic pain, Chen and Meng summarize the evidence of BoNT-A treatment for CPPS in animal and clinical studies in female patients [12].

BoNT-A has also been enthusiastically applied in the treatment of voiding dysfunction due to a neurogenic or non-neurogenic urethral sphincter. Kao et al. review the currently published clinical research on BoNT-A in the context of urethral sphincter dysfunction [13]. Therapeutic efficacy is usually accompanied by adverse events, which limit the wide application of this reasonable treatment for refractory voiding dysfunction. Lee and Kuo report their clinical outcome regarding the use of BoNT-A on voiding dysfunction due to different types of bladder and urethral sphincter dysfunction [14]. They found that about 60% of patients with either neurogenic or non-neurogenic voiding dysfunction can benefit from BoNT-A treatment, but increased urinary incontinence was reported in 13% of patients.

In the application of BoNT-A on prostate diseases, Chen et al. review the animal studies of BoNT-A for CP/CPPS [15]. They show that BoNT-A is able to relieve the pain from CP/CPPS and has the potential to serve as an adjunct treatment in the treatment of CP/CPPS. Hsu et al., in a previous review, examined the application of BoNT-A for patients with LUTS/BPH and clinical data and found that BoNT-A is effective in a subset of patients with clinical BPH [16]. For the inhibition of chronic prostatic inflammation, Chiang et al. review the literature and report that the therapeutic effect of BoNT-A on BPH is limited to a certain subsets of BPH patients with LUTS, especially in men with concomitant CP/CPPS [17]. Finally, Chang et al. compare the efficacy and safety of the mid-urethral sling (MUS) with and without BoNT-A in women with mixed urinary incontinence only. They found that BoNT-A injection with the MUS demonstrates efficacy and safety in the treatment of mixed urinary incontinence, specifically for women with DO. [18]. With the careful selection of patients suitable for BoNT-A injection, this treatment modality might be applied to several LUTDs that are difficult to manage by currently available medication.

This Special Issue of *Toxins* covers the pathophysiology and therapeutic potential of BoNT-A for LUTD, with emphasis on the mechanism of pharmacological action and clinical effects. Readers can learn the basic science and clinical therapy of BoNT-A in the treatment of refractor OAB, DO, IC/BPS, LUTS/BPH, and CP/CPPS, as well as voiding dysfunction.
